# Adiponectin regulates bone mass in AIS osteopenia via RANKL/OPG and IL6 pathway

**DOI:** 10.1186/s12967-019-1805-7

**Published:** 2019-02-28

**Authors:** Hong-qi Zhang, Long-jie Wang, Shao-hua Liu, Jiong Li, Li-ge Xiao, Guan-teng Yang

**Affiliations:** Department of Spine Surgery, Xiangya Hospital of Central-South University, Changsha, 410008 Hunan China

**Keywords:** Adiponectin, Adiponectin receptor 1, Adolescent idiopathic scoliosis, Osteopenia, RANKL, OPG, IL6

## Abstract

**Background:**

Osteopenia have been well documented in adolescent idiopathic scoliosis (AIS). Adiponectin has been shown to be inversely proportional to body mass index and to affect bone metabolism. However, the circulating levels of adiponectin and the relationship between adiponectin and low bone mass in AIS remain unclear.

**Methods:**

A total of 563 AIS and 281 age-matched controls were recruited for this study. Anthropometry and bone mass were measured in all participants. Plasma adiponectin levels were determined by enzyme-linked immunosorbent assay (ELISA) in the AIS and control groups. An improved multiplex ligation detection reaction was performed to study on single nucleotide polymorphism. Facet joints were collected and used to measure the microstructure, the expression of RANKL, OPG, osteoblast-related genes, inflammatory factors, adiponectin and its receptors by qPCR, western blotting and immunohistochemistry. Furthermore, primary cells were extracted from facet joints to observe the reaction after adiponectin stimulation.

**Results:**

Compared with the controls, lower body mass index and a marked increase in circulating adiponectin were observed in AIS osteopenia (17.09 ± 1.09 kg/m^2^ and 21.63 ± 10.30 mg/L). A significant difference in the presence of rs7639352 was detected in the AIS osteopenia, AIS normal bone mass and control groups. The T allele showed a significant higher proportion in AIS osteopenia than AIS normal bone mass and control groups (41.75% vs 31.3% vs 25.7%, p < 0.05). micro-CT demonstrated that the AIS convex side had a significant lower bone volume than concave side. RNA and protein analyses showed that in cancellous bone, higher RANKL/OPG and adipoR1 levels and lower runx2 levels were observed, and in cartilage, higher adipoR1 and IL6 levels were observed in AIS. Furthermore, convex side had higher RANKL/OPG, IL6 and adipoR1 than concave side. Compared with normal primary cells, convex side primary cells showed the most acute action, and concave side primary cells showed the second-most acute action when exposed under same adiponectin concentration gradient.

**Conclusion:**

Our results indicated that high circulating adiponectin levels may result from gene variations in AIS osteopenia. Adiponectin has a negative effect on bone metabolism, and this negative effect might be mediated by the ADR1-RANKL/OPG and ADR1-IL6 pathways.

**Electronic supplementary material:**

The online version of this article (10.1186/s12967-019-1805-7) contains supplementary material, which is available to authorized users.

## Background

Adolescent idiopathic scoliosis (AIS) is a complex 3-dimensional spinal deformity of unknown cause occurring in adolescents between 10 years old and 18 years old. It affects approximately 5% of all children and mostly girls, with a ratio of 10 girls to each boy [1]. Although brace treatment can significantly decrease the progression of the curves, 28% of AIS patients demonstrate curve progression even after bracing [2]. Severe scoliosis can evolve to the chest and rib, causing cardiac dysfunction, pulmonary constraints and back pain. Generally, surgery is the only way to treat severe curves, which may cause loss of spinal mobility and quality of life.

There is no widely agreed etiology of AIS. Several theories on the pathogeny of AIS strongly indicate that AIS is a multifactorial disease that involves genetic factors; hormones; and neuromuscular, environmental and lifestyle factors [3–5]. Moreover, an abnormal growth pattern has been well documented in AIS, in particular, a low body mass index and systemic low bone mass. Since the Burner group first reported the association of low bone mineral density in AIS in 1982, scholars have focused on the relationship between osteopenia and AIS [6]. Most researchers have found that the incidence rate of osteopenia, which is the most common metabolic bone disease in adults but is rare in adolescents, is much higher in AIS than in adolescent populations, ranging from 20 to 38% [7, 8]. In addition, osteopenia is considered one of the causes of scoliosis curvature progression and curve severity [9, 10]. Patients who have osteopenia may have a larger Cobb angle, faster progression, and a worse outcome of brace treatment. However, the cause of osteopenia in AIS remains unclear, and whether the low bone mass is a primary or secondary cause is still under investigation. Smith suggested that eating behavior may be a contributing factor [11], whereas Lee implied that a low calcium intake may influence bone mass [12]. Cheung et al. [13] found that abnormal bone mineralization and failure to catch up with increased bone growth during the peripubertal period lead to osteopenia. Some studies have indicated that AIS is a systemic disease and metabolic abnormalities occur in the disease. Abnormal levels of estrogen, leptin and melatonin have been found in AIS and might play an important role in osteopenia [14, 15].

Adiponectin (also known as Acrp 30, AdipoQ) is a 30-kd protein secreted by adipocytes that has received much attention in obesity with respect to regulating insulin sensitivity, energy balance and cellular metabolism [16]. Its serum level ranges from 3 to 30 µg/mL and is inversely proportional to body fat in both sexes [17]. Furthermore, adiponectin is the most abundant circulating adipokine and exerts widespread beneficial metabolic effects via its cell surface receptors (adipoR1 and adipoR2), which contain seven transmembrane domains differing from those of G protein-coupled receptors. In addition, adiponectin and its receptors are found in bone-forming cells and bone marrow cells, which suggests a cross-regulatory link between adipose tissue and bone metabolism [18, 19]. In vitro, adiponectin induces human osteoblast proliferation and differentiation, promotes RANKL expression and inhibits OPG expression through the mitogen-activated protein kinase (MAPK) pathway [20, 21]. Other scholars have reported that adiponectin increases bone mass by suppressing osteoclastogenesis and activating osteoblastogenesis [22]. In vivo, 4-week-old adiponectin-knockout mice exhibit retarded bone growth, reduced trabecular bone volume, decreased cortical bone, and an increased osteoclast number [23]. Another study revealed that 12-week-old adiponectin^−/−^ mice show a high bone mass affecting the axial and appendicular skeleton and trabecular and cortical bones, and transgenic mice harboring a fivefold increase in adiponectin circulating levels display a low bone mass phenotype [24]. Various clinical studies have also reported that circulating adiponectin levels are negatively correlated with bone mineral density in premenopausal and postmenopausal women along with young and elderly men, and the risk of fracture increases with increasing serum adiponectin levels, at least in men [25–27].

Although previous research has shown contradictory results for bone metabolism, there is no research focusing on adiponectin in the regulation of bone mass in AIS. Because individuals with AIS often exhibit a low body mass index and a low systemic bone mass, based on the intriguing, prior research, we explored adiponectin in AIS to determine what effect it has on AIS pathogenesis. In this study, adiponectin plasma levels were determined by enzyme-linked immunosorbent assay (ELISA), and the expression levels of adiponectin and its receptors were tested in the facet joint of apical vertebrae in AIS. Primary cells of patients were extracted to explore whether these cells are influenced by adiponectin.

## Methods

### Ethics statement

This study was approved by the Ethics Committee of Xiangya Hospital of Central South University (No. 201703358). Informed written consent was signed by all subjects and their legal guardians before participation in the study.

### Subjects

All subjects were collected between September 1, 2016, and May 20, 2018, including ELISA subjects, single nucleotide polymorphism (SNP) subjects, micro-CT subjects and surgical specimen subjects. All subjects were screened through a detailed questionnaire, medical history, physical examination including an Adams forward bending test, and an imaging examination including X-rays, computerized tomography and magnetic resonance imaging. Diagnoses of the patients were also confirmed by three experienced surgeons. For AIS enrollment, the inclusion criteria were a minimum Cobb angle of 10°; a complete clinical data record including age, gender, Cobb angle, Risser sign, height, weight, body mass index (BMI), bone marrow density (BMD), Z score and Lenke classification. For controls enrollment, the inclusion criteria were the age-matched; non-AIS controls including lumbar herniation, spine fracture and healthy people in our Medical examination center; a complete clinical data record including age, gender, Risser sign, height, weight, body mass index (BMI), bone marrow density (BMD) and Z score. The exclusion criteria for the AIS and controls included endocrine diseases; metabolic disorders; skeletal dysplasia; scoliosis secondary to neuromuscular diseases, congenital vertebral malformations and other etiology; connective tissue abnormalities; disorders affecting bone metabolism; history of recent medicine intake; and history of spinal surgery. The ELISA subjects comprised 92 AIS subjects aged 10–18 years and 35 age-matched controls (Table 1). For SNP subjects, a total of 409 AIS patients and 206 controls were recruited from our spine center and medical examination center (Additional file 1: Table S1). Moreover, among the AIS patients, two group were divided according to the bone mass. For micro-CT subjects, facet joints of apical vertebrae were obtained from 5 AIS (Additional file 2: Table S2). 30 AIS and 30 controls were enrolled in real-time quantitative pcr and western blotting (Additional file 3: Table S3). And for immunochemistry and TRAP staining, 22 AIS and 15 controls were recruited (Additional file 4: Table S4). For cell experiments, facet joints of apical vertebrae were obtained from 5 AIS and 5 lumbar disc herniations (Additional file 5: Table S5).

**Table 1:** Clinical data of ELISA subjects

Items	AIS group			Control	p value
	Osteopenia	Normal bone mass	Total		
Number (male/female)	42 (24/18)	50 (23/27)	92 (47/45)	35 (24/11)	0.117
Age (years)	14.26 ± 2.28	13.5 ± 2.12	13.85 ± 2.22	14.31 ± 2.04	0.137
Weight (kg)	41.63 ± 7.45	43.3 ± 7.92	42.54 ± 7.72	48.34 ± 7.32	0.001
Height (m)	1.55 ± 0.12	1.56 ± 0.13	1.56 ± 0.13	1.60 ± 0.12	0.259
BMI (kg/m^2^)	17.01 ± 1.05	17.54 ± 1.16	17.30 ± 1.14	18.76 ± 1.24	< 0.05
Risser sign	2.17 ± 1.67	1.78 ± 1.75	1.96 ± 1.72	2.34 ± 1.70	0.257
Lumbar spine					
LSBMC (g)	33.33 ± 8.67	40.58 ± 11.18	37.28 ± 10.70	44.56 ± 14.09	< 0.05
LSBMD (g/m^2^)	0.67 ± 0.11	0.80 ± 0.13	0.74 ± 0.14	0.86 ± 0.13	< 0.05
LS Z score	− 1.98 ± 0.84	− 0.27 ± 0.45	− 1.05 ± 1.08	− 0.22 ± 0.52	< 0.05
Femoral neck					
FNBMC (g)	18.87 ± 5.94	20.75 ± 6.28	19.90 ± 6.17	26.79 ± 9.80	< 0.05
FNBMD (g/cm^2^)	0.66 ± 0.12	0.73 ± 0.10	0.70 ± 0.11	0.84 ± 0.14	< 0.05
FN Z score	− 2.3 ± 0.87	− 1.34 ± 0.59	− 1.8 ± 0.86	− 0.7 ± 0.95	< 0.05
Adiponectin (mg/L)	21.63 ± 10.30	11.66 ± 6.96	16.21 ± 9.94	8.66 ± 7.53	< 0.01
Main curve cobb angle (°)	25.520 ± 8.06	20.52 ± 4.95	22.80 ± 6.98		
Lenke classification					
I	18	22	40		
II	1	3	4		
III	3	5	8		
IV	1	0	1		
V	15	16	31		
VI	4	4	8		

One way ANOVA was use to tested, when p value > 0.05, there was no significant difference in all the groups; when p value < 0.05, then using bonferroni correction to test each two groups. The p value in this table was represent difference among three groups

### Anthropometric and BMD assessment

Anthropometric measurements, including age, body weight, standing height, risser sign, main curve cobb angle and lenke classification were recorded in all patients. BMI was computed by dividing weight (kg) by height squared (m^2^). Lumbar spine BMD and femoral neck BMD were measured by dual-energy X-ray absorptiometry in all AIS and controls. In general, the T-score was used to distinguish osteopenia and osteoporosis from normal bone density in postmenopausal women, whereas in puberty, when the peak bone mass did not reach the peak value, the age-adjusted Z score was used to identify osteopenia and osteoporosis. The Z score was used for comparison to the age-matched normal group, and a Z score higher than − 1 standard deviation was classified as normal bone mass; a Z score less than − 1 standard deviation was classified as osteopenia.

### Isolation and culture of human primary osteoblasts

During posterior spinal fusion, facet joints of the apical vertebrae were harvested and divided into two parts (5 AIS patients and 5 controls subjects). Cartilage was peeled from articular facets, and cancellous bone was isolated from the remaining parts under sterile conditions. The cartilage was washed with phosphate buffer solution (PBS) three times, followed by 0.25% trypsin (Gibco, Carlsbad, CA, USA) treatment for 30 min in an incubator (5% CO_2_ atmosphere at 37 °C). After the digestion was completed, a serum-containing culture medium was added to terminate the digestion. The supernatant was removed after centrifugation, and the precipitate was resuspended in 0.1% collagenase type II (Sigma-Aldrich, St. Louis, MO, USA) and placed for 4 h in an incubator. Finally, the fragment was plated on a 6-well culture plate in DMEM/high-glucose medium (Gibco, Carlsbad, CA, USA) containing 10% FBS (Gibco, Carlsbad, CA, USA) and 1% P/S (Gibco, Carlsbad, CA, USA) in the incubator. The medium was renewed every 3–4 days. The cancellous bone was also washed with PBS and digested with 0.25% trypsin for 30 min. After the digestion was completed, 0.1% collagenase type I was added to the fragment for 4 h in the incubator. Finally, the fragment was plated on a 6-well culture plate in DMEM/F12 (Gibco, Carlsbad, CA, USA) medium containing 10% FBS, 1% P/S in the incubator.

### Alizarin red staining

P2 generation osteoblasts were inoculated in 6-well culture plates. After being cultured for 28 days, the cells were fixed with anhydrous alcohol for 10 min, incubated with 0.1% Alizarin Red S (Sigma) for 10 min, and then washed with distilled water three times. Finally, the cells was dehydrated with graded ethanol and observed under the microscope.

### Toluidine blue staining

P2 generation chondrocytes were inoculated in 24-well culture plates with coverslips. After reaching 80% confluence, 4% paraformaldehyde (PFA) was applied for 20 min to fix the cell and washed with PBS three times. Then, the cells were incubated with 0.05% toluidine blue for 1 h and then washed with distilled water three times. Finally, the cells were washed with 70% ethanol 2 times and PBS 3 times and then observed under the microscope.

### Trap staining

The paraffin section was placed in xylene twice for 20 min each time and rehydrated with 100% alcohol twice each for 5 min and 75% alcohol for 5 min. After washing with double-distilled water 3 times, the sections were stained with tartrate-resistant acid phosphatase (TRAP) using a commercially available kit (Servicebio, China). TRAP^+^ multinucleated cells (MNCs) showing more than three nuclei were considered to be osteoclasts.

### Real-time quantitative PCR

Facet joints were divided into two parts to extract RNA (30 AIS patients and 30 controls). Cartilage and cancellous bone were separated from the facet joints and cut into pieces (1 mm^3^) with a sterile scalpel on silver paper. The cartilage and cancellous tissues were flash-frozen in liquid nitrogen and homogenized with a traditional mortar or pestle. Then, total RNA was extracted with TRIzol reagent (Invitrogen, USA) according to the manufacturer’s instructions. cDNA was synthesized from total RNA using a HiFiScript cDNA Synthesis Kit (CWBIO, China). Real-time PCR was performed using SYBR qPCR SuperMix Plus (NovoStart, China) and an Applied Biosystems 7500 instrument. The following primers were used for quantitative PCR: RANKL: TCCCATCTGGTTCCCATAAA (forward) and CTTGGGATTTTGATGCTGGT (reverse); OPG: GGCAACACAGCTCACAAGAA (forward) and CGGTAAGCTTTCCATCAAGC (reverse); RANK: TTGCAGCTCAACAAGGACAC (forward) and AGCTGGCAGAGAGAAGAACTGC (reverse); Osterix: GCTTATCCAGCCCCCTTTAC (forward) and CACTGGGCAGACAGTCAGAA (reverse); ALP: GGTGAACCGCAACTGGTACT (forward) and TCTGGGTACTCAGGGTCTGG (reverse); RUNX2: TGTTTGGCGACCATATTGAA (forward) and GGCTGCAAGATCATGACTGA; ADIPONECTIN: GCTGGGAGCTGTTCTACTGC (forward) and CGATGTCTCCCTTAGGACCA (reverse); ADR1: CCTTCTACTGCTCCCCACAG (forward) and GACAAAGCCCTCAGCGATAG (reverse); ADR2: TGAAGGTCCATTCTCCCAAG (forward) and CAAATCTCCTTGGTGGCTGT (reverse); IL6: AGGAGACTTGCCTGGTGAAA (forward) and CAGGGGTGGTTATTGCATCT (reverse); IL10: AAGCCTGACCACGCTTTCTA (forward) and ATGAAGTGGTTGGGGAATGA (reverse); TNF a: TCCTTCAGACACCCTCAACC (forward) and AGGCCCCAGTTTGAATTCTT (reverse); 18S: AGAAACGGCTACCACATCCA (forward) and CCCTCCAATGGATCCTCGTT (reverse); The RNA levels were normalized by 18 s. The program was 95 °C for 1 min and 40 cycles including 95 °C for 20 s and 60 °C for 1 min.

### Western blotting

For protein extraction, facet joints were divided into cartilage and cancellous bone and cut into pieces (1 mm^3^) on silver paper (30 AIS patients and 30 control subjects). The cartilage and cancellous tissues were flash-frozen in liquid nitrogen and homogenized with a traditional mortar or pestle. Then, total protein was extracted with ice cold RIPA Lysis Buffer reagent (CWBIO, China) including PMSF and a phosphatase inhibitor, according to the manufacturer’s instructions. The protein levels were quantified using a bicinchoninic acid (BCA) protein assay reagent (Beyotime, China). Fifteen micrograms of protein for each well were separated by 10% SDS gel and then transferred to a PVDF membrane. The membrane was blocked with defatted milk for 1 h at room temperature, followed by incubation with a primary antibody at 4 °C on a rocker overnight. Primary antibodies included adiponectin receptor 1 (Abcam, USA), RANKL (Abcam, USA), OPG (Abcam, USA), RANK (abcam, USA), adiponectin receptor 1 (Abcam, USA). After incubation with secondary antibodies for 1 h at room temperature, BeyoECL Plus (Beyotime, China) was used to detect the blot. The protein levels were normalized by total protein using TGX FastCast (Bio-rad, USA).

### Immunochemistry

Facet joint tissues were obtained from AIS patients and controls during surgery (22 AIS patients and 15 control subjects). Then, the tissues were formalin fixed, decalcified and paraffin embedded. The paraffin sections were placed in xylene twice and rehydrated with graded alcohol (100%, 95, 85%, 70% alcohol each for 1 min, and double distilled water for 2 min), followed by treatment with 2% hydrogen peroxide for 10 min. After incubation with 5% bovine serum albumin (BSA) for 30 min at room temperature, primary antibodies were added to paraffin sections at 37 °C overnight. Primary antibodies included adiponectin receptor 1 (Abcam, USA) and OCN (Servicebio, China). Then, the sections were incubated with biotinylated goat anti-rabbit immunoglobulins for 30 min, followed by incubation with streptavidin–horseradish peroxidase solution (ZSGB-BIO, China) for 30 min, which bound to the secondary antibody. After washing 3 times with PBS, the sections were stained with 3,3′-diaminobenzidine (DAB) for 30 s. Then, the sections were washed in water, counterstained with hematoxylin (Servicebio, China) for 2 min and observed under the microscope.

### Micro-CT

Facet joints were harvested from 5 AIS patients during surgery. Scanning was performed at 70 kV, 200 μA, 20 μm per pixel and a fixed exposure time of 300 ms using a μCT100 micro-CT scanner (Scanco Medical, Bruttisellen, Switzerland). Evaluation V6.5-3 was used as image reconstruction software for further analyses. Bone volume per tissue volume (BV/TV), trabecular thickness (Tb.Th), trabecular number (Tb.N) and trabecular separation (Tb.Sp) were measured.

### ELISA

Peripheral venous blood samples were collected from all patients and controls in the morning after overnight fasting, and the blood samples were drawn into an EDTA tube. The samples were immediately delivered to the laboratory, centrifuged for 10 min at 1000×*g* at 4 °C and stored at − 80 °C until batch analysis. Before the test, the plasma sample should be dilute with sample diluent (1:500) according to the manufacture. Diluted sample was quantified by ELISA (Cusabio Biotech, Wuhan, China) with a detection in adiponectin ranging from 1.562 to 100 ng/mL.

### Genotyping

Genomic DNA was extracted from peripheral blood using an SQ Blood DNA Kit II (OMEGA BIO-TEK, America). The SNP genotyping work was performed using an improved multiplex ligation detection reaction (iMLDR) technique developed by Genesky Biotechnologies, Inc. (Shanghai, China). For each SNP, the alleles were distinguished by different fluorescent labels of allele-specific oligonucleotide probe pairs. Different SNPs were further distinguished by different extended lengths at the 3′ end. Two negative controls were set: one with double-distilled water as the template and the other with a DNA sample without primers while keeping all other conditions the same in one plate. Duplicate tests were designed, and the results were consistent. A random sample accounting for ~ 5% of the total DNA samples was directly sequenced using Big Dye-terminator version 3.1 and an ABI3730XL automated sequencer (Applied Biosystems) to confirm the results of iMLDR.

### Statistics

Results were recorded and analyzed by SPSS software (version 24.0; SPSS, Inc., Chicago, IL, USA). In the genetic association study, the Hardy–Weinberg equilibrium (HWE) test was performed, and allelic association analyses were performed by using Chi square tests and Bonferroni correction. Quantitative data are expressed as the mean ± standard deviation and were assessed by one-way ANOVA, Bonferroni correction and T-tests. The difference was considered significant if the p value was < 0.05 and Bonferroni correction showed significant difference when the p value was < 0.0167.

## Results

The results of the study are presented in three parts. In the first part, patients with AIS were divided into two groups on the basis of bone mass. Plasma adiponectin levels were measured in the AIS and control groups. Next, 409 subjects with AIS and 206 controls were recruited to genotype 9 SNPs that may affect adiponectin serum levels. In addition, AIS patients were also divided into two groups on the basis of bone mass. In the second part, morphology of apical vertebra facet joints was studied and osteoclasts, osteoblasts related genes, inflammatory factor, adiponectin and its receptors were test by qPCR, western blotting and immunohistochemistry. In the third part, to investigate the exact mechanism of how adiponectin affects bone mass, primary cells were extracted from facet joints to observe the reaction after adiponectin stimulation.

### Serum level of adiponectin in low bone mass AIS, normal bone mass AIS and control samples

**Fig. 1:**
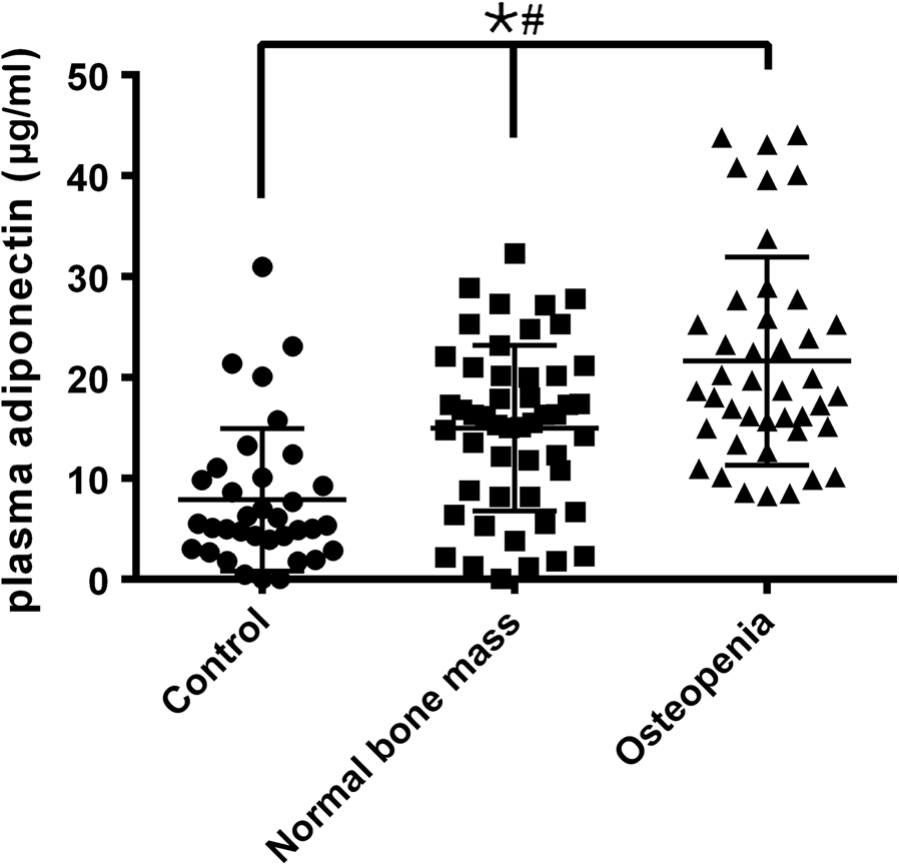
Plasma adiponectin level in AIS osteopenia, AIS normal bone mass and control. The plasma adiponectin level were measured by ELISA Kit in AIS osteopenia, AIS normal bone mass and control groups. One way ANOVA was use to tested, when p value > 0.05, there was no significant difference in all the groups; when p value < 0.05, then using Bonferroni correction to test each two groups. *p < 0.05 Bonferroni correction test between controls and AIS osteopenia. ^#^p < 0.0167 Bonferroni correction test between AIS osteopenia and AIS normal bone mass. There is no significant difference between controls and AIS normal bone mass

To assess the plasma adiponectin level, a total of 92 AIS patients and 35 age-match controls were enrolled in the study. The AIS group was divided into two groups on the basis of BMD. The clinical data of the patients and controls are listed in Table 1. There was no significant difference in the ratio of males to females, age, height, or Risser sign between the AIS and control groups. However, AIS patients exhibited a lower BMI (17.51 ± 1.26 vs 18.49 ± 1.27 kg/m^2^, p < 0.01) and a lower weight (40.15 ± 5.65 vs 43.6 ± 4.90 kg, p < 0.05) than did the control group. For AIS osteopenia patients, a significantly lower BMI (17.09 ± 1.19 vs 17.86 ± 1.22 kg/m^2^, p < 0.01) and a larger curve Cobb angle (25.52 ± 8.06° vs 20.52 ± 4.95°, p < 0.01) were observed when compared with those of patients with normal-bone-mass AIS. ELISA showed that adiponectin was normally distributed in both AIS and control subjects (Fig. 1). However, AIS showed a significantly higher adiponectin level (16.21 ± 9.94 vs 8.66 ± 7.53 mg/L, p < 0.01). Osteopenia AIS had a higher adiponectin level than did normal-bone-mass AIS (21.63 ± 10.30 vs 11.66 ± 6.96 mg/L, p < 001). In addition, there was no significant difference between the normal-bone-mass group and the control group (p = 0.062).

### SNP genotype frequency distributions in AIS patients and control group

We performed a nine SNP genotyping which were previously shown to be related to serum adiponectin level in AIS and control groups. A total of 409 AIS and 206 controls were successfully genotyped and subjected to statistical analysis. Among the AIS, patients were divided into two groups on the basis of bone mass. 200 AIS osteopenia were defined as group A (male/female: 83/117, age: 15.35 ± 2.53), 209 AIS normal bone mass (male/female: 99/110, age: 15.05 ± 1.95) were defined as group B and 206 controls (male/female: 105/101, age: 15.52 ± 2.1) were defined as group C. There were no significant differences in age or gender among the groups. Other clinical data are listed in Additional file 1: Table S1.

Nine SNPs genotyped in three groups conformed to the Hardy–Weinberg equilibrium analysis (p > 0.05). Genotype and allele distribution frequencies are listed in Table 2. A significant difference in the presence of rs7639352 was detected in the three groups (p < 0.05). Overall, the frequencies of the C/T and T/T genotype in the Group A were significantly higher than those in the Group B and C (C/T genotype: 51.5% vs 46.4% vs 34%, p < 0.05; T/T genotype: 16% vs 8.1% vs 8.7%, p < 0.05). When compared with each two groups, significant difference was observed between group A and B (p_A–B_ = 0.006), group A and C (p_A–C _< 0.0167). And there is no significant difference between group B and C (p_B–C_ = 0.032). And T allele showed a significant higher proportion in group A than other two group (41.75% vs 31.3% vs 25.7%, p< 0.05).

**Table 2:** Distribution frequencies AIS osteopenia, AIS normal bone mass and control groups

Single nucleotide polymorphism (associate gene)	Group A (AIS osteopenia)	Group B (AIS normal bone mass)	Group C (control)	p_A–B–C_	p_A–B_	p_A–C_	p_B–C_
	Number (%)	Number (%)	Number (%)				
rs7639352 (ADIPOQ)							
C/C	65 (32.5%)	95 (45.5%)	118 (57.3%)	< *0.05*	*0.006*	< *0.0167*	0.032
T/T	32 (16%)	17 (8.1%)	18 (8.7%)				
C/T	103 (51.5%)	97 (46.4%)	70 (34%)				
Allele							
C	233 (58.25%)	287 (68.7%)	306 (74.3%)	< *0.05*	*0.002*	< *0.0167*	0.074
T	167 (41.75%)	131 (31.3%)	106 (25.7%)				
rs10937273 (ADIPOQ)							
G/G	72 (36%)	70 (33.5%)	72 (35%)	0.957			
A/A	33 (16.5%)	33 (15.8%)	36 (17.5%)				
G/A	95 (47.5%)	106 (50.7%)	98 (47.6%)				
rs1648707 (ADIPOQ)							
C/C	50 (25%)	42 (20.1%)	48 (23.3%)	0.588			
A/A	51 (25.5%)	62 (29.7%)	64 (31.1%)				
C/A	99 (49.5%)	105 (50.2%)	94 (45.6%)				
rs266719 (ADIPOQ)							
C/C	154 (77%)	167 (79.9%)	156 (75.7%)	0.355			
T/T	3 (1.5%)	2 (1%)	0 (0%)				
C/T	43 (21.5%)	40 (19.1%)	50 (24.3%)				
rs822354 (ADIPOQ)							
G/G	150 (75%)	150 (71.8%)	140 (68%)	0.585			
A/A	3 (1.5%)	5 (2.4%)	4 (1.9%)				
G/A	47 (23.5%)	54 (25.8%)	62 (30.1%)				
rs822387 (ADIPOQ)							
T/T	199 (99.5%)	208 (99.5%)	206 (100%)	0.603			
C/T	1 (0.5%)	1 (0.5%)	0				
rs17300539 (ADIPOQ)							
G/G	200 (100%)	209 (100%)	206 (100%)	1			
rs12342 (ADIPOQ RECEPTOR 2)							
C/C	51 (25.5%)	45 (21.5%)	56 (27.2%)	0.236			
T/T	43 (21.5%)	61 (29.2%)	58 (28.2%)				
C/T	106 (53%)	103 (49.3%)	92 (44.7%)				
rs11642015 (FTO)							
C/C	156 (78%)	159 (76.1%)	150 (72.8%)	0.801			
T/T	4 (2%)	4 (1.9%)	4 (1.9%)				
C/T	40 (20%)	46 (22%)	52 (25.2%)				

Chi square test was use to tested, when p value > 0.05, there was no significant difference in all the groups; when p value < 0.05, then using bonferroni correction to test each two groups. p_A–B–C_ represents difference among three groups. p_A-B_ represents difference between group A and B. p_A–C_ represents difference between group A and C. p_B–C_ represents difference between group B and C. If p value < 0.0167, there was significant difference between two groups. The italic values represent the significant difference

### Morphology of apical vertebra facet joints

Facet joints were obtained from 5 patients and the clinical data are listed in Additional file 2: Table S2. Micro-CT analysis revealed that the trabecular bone volume (BV/TV) in AIS convex was significantly lower than concave. Difference % was defined as: (AIS convex–AIS concave)/AIS concave. The mean difference % in BV/TV, Tb.N, Tb.Th, Tb.Sp were − 0.15, − 0.11, 0.019 and 0.23, respectively (Table 3). And there were significant difference in BV/TV (p = 0.0061), Tb.N (p = 0.0017) and Tb.Sp (p = 0.018). These results showed that AIS convex side bone volume was significant lower than concave side.

**Table 3:** Bone microstructure with micro-CT in AIS concave and convex side

Items	Position	Convex/concave	BV/TV	Tb.N	Tb.Th	Tb.Sp
Patients 1	T9	Convex	0.34	1.64	0.24	0.62
		Concave	0.46	1.77	0.29	0.55
	L2	Convex	0.32	1.89	0.24	0.55
		Concave	0.39	2.06	0.25	0.48
Patients 2	L2	Convex	0.6	1.92	0.49	0.50
		Concave	0.8	2.5	0.48	0.32
Patients 3	T8	Convex	0.56	2.44	0.39	0.38
		Concave	0.62	2.98	0.30	0.25
Patients 4	T8	Convex	0.45	1.99	0.30	0.47
		Concave	0.53	2.15	0.31	0.44
Patients 5	T9	Convex	0.36	1.48	0.25	0.67
		Concave	0.34	1.55	0.25	0.67
Difference %			− 0.15	− 0.11	0.019	0.23
p value			0.0061	0.0017	0.42	0.018

BV/TV is bone volume fraction; Tb.N is trabecular number; Tb.Th is trabecular thickness; Tb.Sp is trabecular separation. Difference  %: (AIS convex–AIS concave)/AIS concave%

The expression levels of osteoclasts, osteoblast-related genes, inflammatory factors, adiponectin and its receptors.

Human facet joints were obtained from the AIS and control groups during surgery. Clinical data was listed in Additional file 3: Table S3 and Additional file 4: Table S4. AIS was divided into two group which were concave group and convex group. Cartilage and cancellous bone were separated from facet joints. RNA and protein were extracted from each tissue to examine the expression of osteoblast and osteoclast marker genes, inflammatory factor, adiponectin and its receptors in the two groups.

**Fig. 2:**
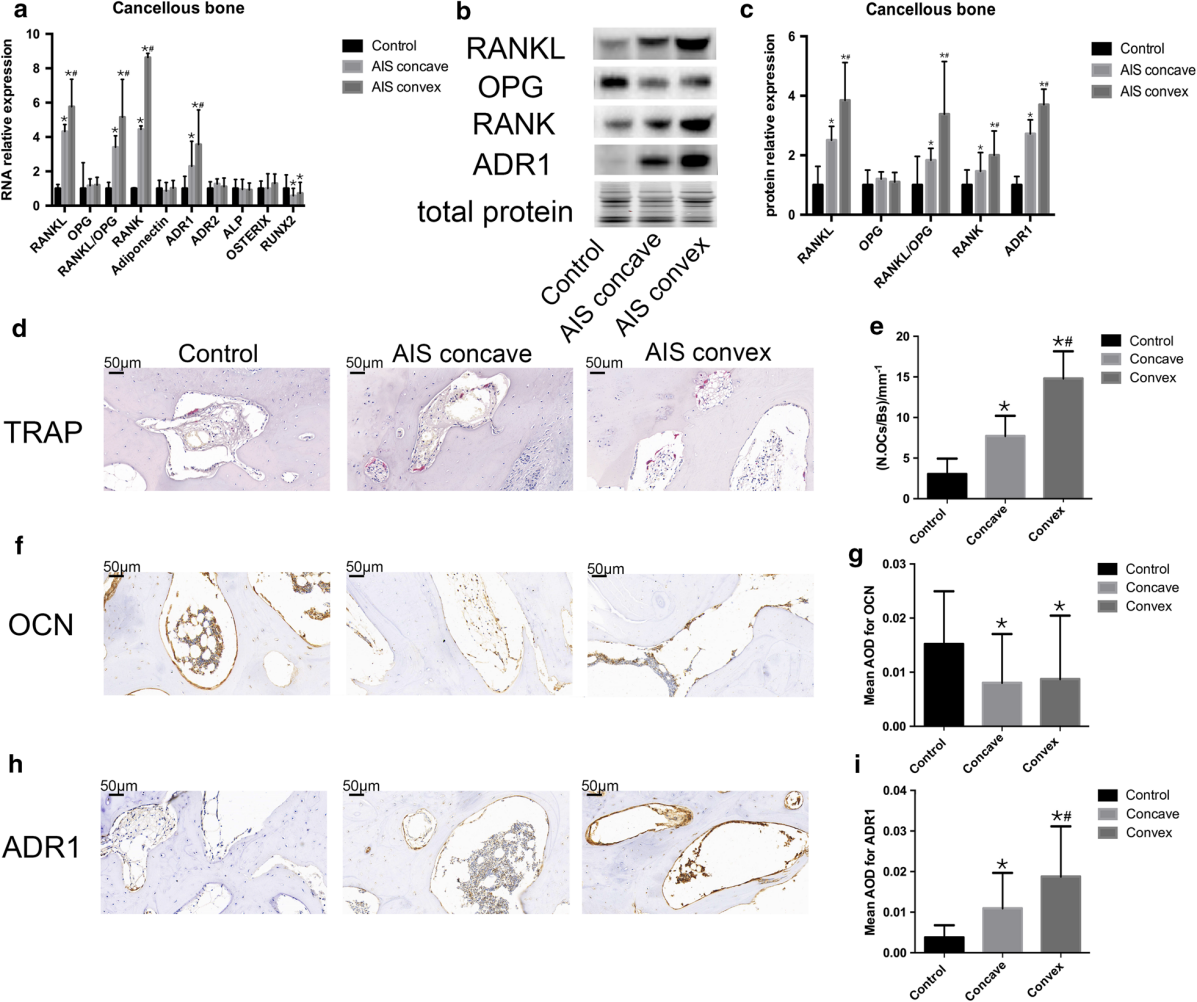
Unbalance between osteoclast and osteoblast and high adipoR1 expression were observed in AIS cancellous bone. **a** Total RNA were extracted from two group. The relative expression level of RANKL, OPG, RANK, adiponectin, adipoR1, adipoR2, ALP, osterix, runx2 were detected by quantitative real-time PCR. The Y axis represents the fold change in transcript levels in three group. The control group was set to 1.0. **b**, **c** Protein level of RANKL, OPG, RANK, adipoR1 were measured by western blotting. The Y axis represents the fold change and control were set to 1.0. **d** TRAP staining of facet joint in AIS and control group. Scale bar: 50 μm. **e** Quantitative analysis of the number of TRAP^+^ cells. BS bone surface, OCs osteoclast. **f**, **h** Representative images of immunohistochemical staining for OCN and adipoR1. **g**, **i** Quantitative analysis of the mean intensity for positively stained areas. Data are shown as the mean ± SD. One way ANOVA was use to tested, when p value > 0.05, there was no significant difference in all the groups; when p value < 0.05, then using bonferroni correction to test each two groups. *p < 0.0167 Bonferroni correction test between control and AIS concave. Bonferroni correction test between control and AIS convex. ^#^p < 0.0167 Bonferroni correction test between AIS concave and AIS convex

In the cancellous bone RNA level, as shown in Fig. 2a, there was no significant difference in OPG (0.80 ± 0.39 vs 0.83 ± 0.45 vs 0.69 ± 0.48, p = 0.419), adiponectin (0.87 ± 0.47 vs 1.06 ± 0.45 vs 1.02 ± 0.48, p = 0.252), ALP (1.00 ± 0.59 vs 0.98 ± 0.40 vs 1.06 ± 0.53, p = 0.828), osterix (0.84 ± 0.86 vs 1.15 ± 0.45 vs 0.84 ± 0.44, p = 0.088) and adipoR2 (0.51 ± 0.29 vs 0.45 ± 0.5 vs 0.4 ± 0.29, p = 0.527) in concave side, convex side and control group, respectively. RANKL, RANKL/OPG, RANK and adipoR1 levels in control group were significantly lower than those in two side group. In control group, the RANKL/OPG was 0.51 ± 0.36, whereas RANKL/OPG in concave side and convex side were 1.73 ± 0.067 and 2.63 ± 2.20, respectively. The RANK in control group was 0.031 ± 0.042 and in concave and convex side were 0.14 ± 0.20 and 0.27 ± 0.24, respectively. And the expression of adipoR1 in concave side and convex side were much higher than that in control group (2.33 ± 1.45 vs 3.6 ± 2.03 vs 1.01 ± 0.71, p < 0.05). In addition, the runx2 level was significantly higher in control group than that in AIS two side groups (p = 0.002). When compared the concave side with convex side, higher RANKL, RANKL/OPG, RANK and adipoR1 level were observed in convex side. There was no significant difference in runx2 expression between two sides. In cancellous bone protein level, Fig. 2b, c showed that that there were no significant difference in OPG between control group (1.03 ± 0.50) and AIS group (concave side: 1.24 ± 0.24; convex side: 1.14 ± 0.32). And RANKL, RANKL/OPG, RANK and adipoR1 in control groups (0.70 ± 0.63, 0.80 ± 0.96, 0.66 ± 0.38, 0.51 ± 0.29) were significant lower than AIS groups. When compared the concave and convex side, RANKL (1.77 ± 0.46 vs 2.70 ± 1.27, p < 0.05), RANK (1.12 ± 0.40 vs 1.82 ± 0.61, p < 0.05), RANKL/OPG (1.46 ± 0.40 vs 2.70 ± 1.77, p < 0.05), adipoR1 (1.4 ± 0.47 vs 1.9 ± 0.52, p < 0.05) were significant higher in convex side than concave side. In immunohistochemistry results, Fig. 2d–i showed that control group had lower osteoclast number (3.04 ± 1.9), lower adipoR1 AOD (0.0038 ± 0.0030) and higher OCN AOD (0.015 ± 0.009) than AIS group. And convex side (14.79 ± 3.35, 0.018 ± 0.012) demonstrated higher osteoclast number and adipoR1 than concave side (7.72 ± 2.49, 0.011 ± 0.008). In addition, there is no significant difference in OCN in convex (0.005 ± 0.002) and concave side (0.008 ± 0.009).

**Fig. 3:**
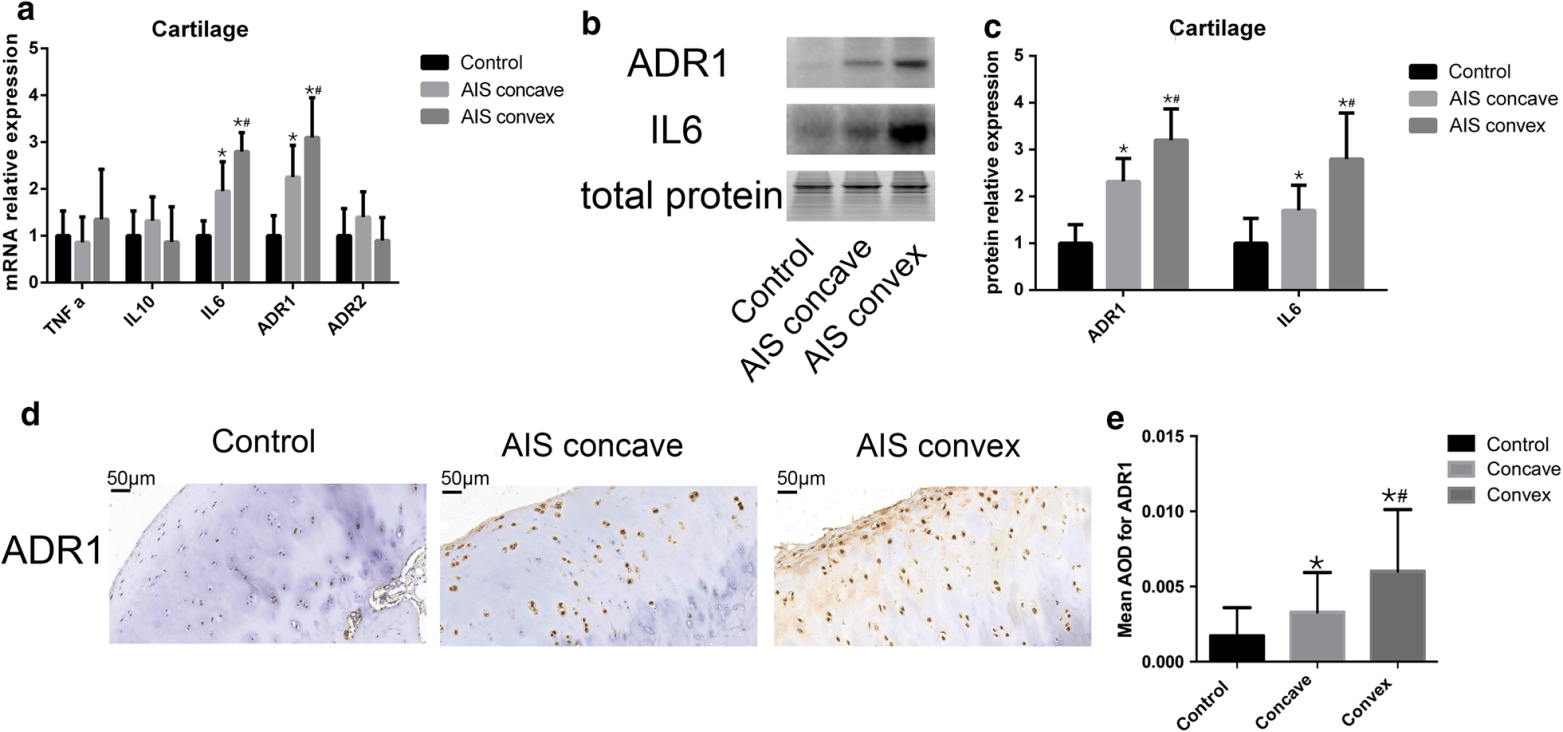
High IL6 and adipoR1 expression were observed in AIS cartilage. **a** Total RNA were extracted from three group. The relative expression level of TNF a, IL10, IL6, adiponectin, adipoR1, adipoR2 were detected by quantitative real-time PCR. The Y axis represents the fold change in transcript levels in three group. The control group was set to 1.0. **b**, **c** Protein level of IL6, adipoR1 were measured by western blotting. The Y axis represents the fold change and control were set to 1.0. **d** Representative images of immunohistochemical staining for adipoR1. **e** Quantitative analysis of the mean intensity for positively stained areas. Data are shown as the mean ± SD. One way ANOVA was use to tested, when p value > 0.05, there was no significant difference in all the groups; when p value < 0.05, then using bonferroni correction to test each two groups. *p < 0.0167 Bonferroni correction test between control and AIS concave. Bonferroni correction test between control and AIS convex. ^#^p < 0.0167 Bonferroni correction test between AIS concave and AIS convex

In cartilage RNA level, TNF a, IL10 and adipoR2 expression showed no significant expression among three group (Fig. 3a). IL6 and adipoR1 levels were significantly higher than those in the controls. In control group, TNF a, IL10, IL6, adipoR1, adipoR2 were 0.61 ± 0.53, 0.98 ± 0.53, 0.60 ± 0.33, 0.54 ± 0.43 and 0.86 ± 0.58, respectively. And when compared the concave side with convex side, higher IL6 and adipoR1 were observed in convex side (1.7 ± 0.4 and 1.69 ± 0.85) than in concave side (1.18 ± 0.63 and 1.22 ± 0.68). In cartilage protein level, Fig. 3b, c shows that IL6 and adipoR1 were significant lower in control group (1.00 ± 0.52, 0.50 ± 0.40) than AIS group. And convex side (2.8 ± 0.98 and 1.6 ± 0.67) showed higher IL6 and adipoR1 than convex side (1.7 ± 0.54 and 1.17 ± 0.49). In immunohistochemistry results, significant lower adipoR1 AOD was observed in control group (0.0017 ± 0.0018) than in AIS group (Fig. 3d, e). And significant higher adipoR1 AOD was observed in convex side (0.0060 ± 0.004) than that in convex side (0.0032 ± 0.0026).

### Cell experiment

**Fig. 4:**
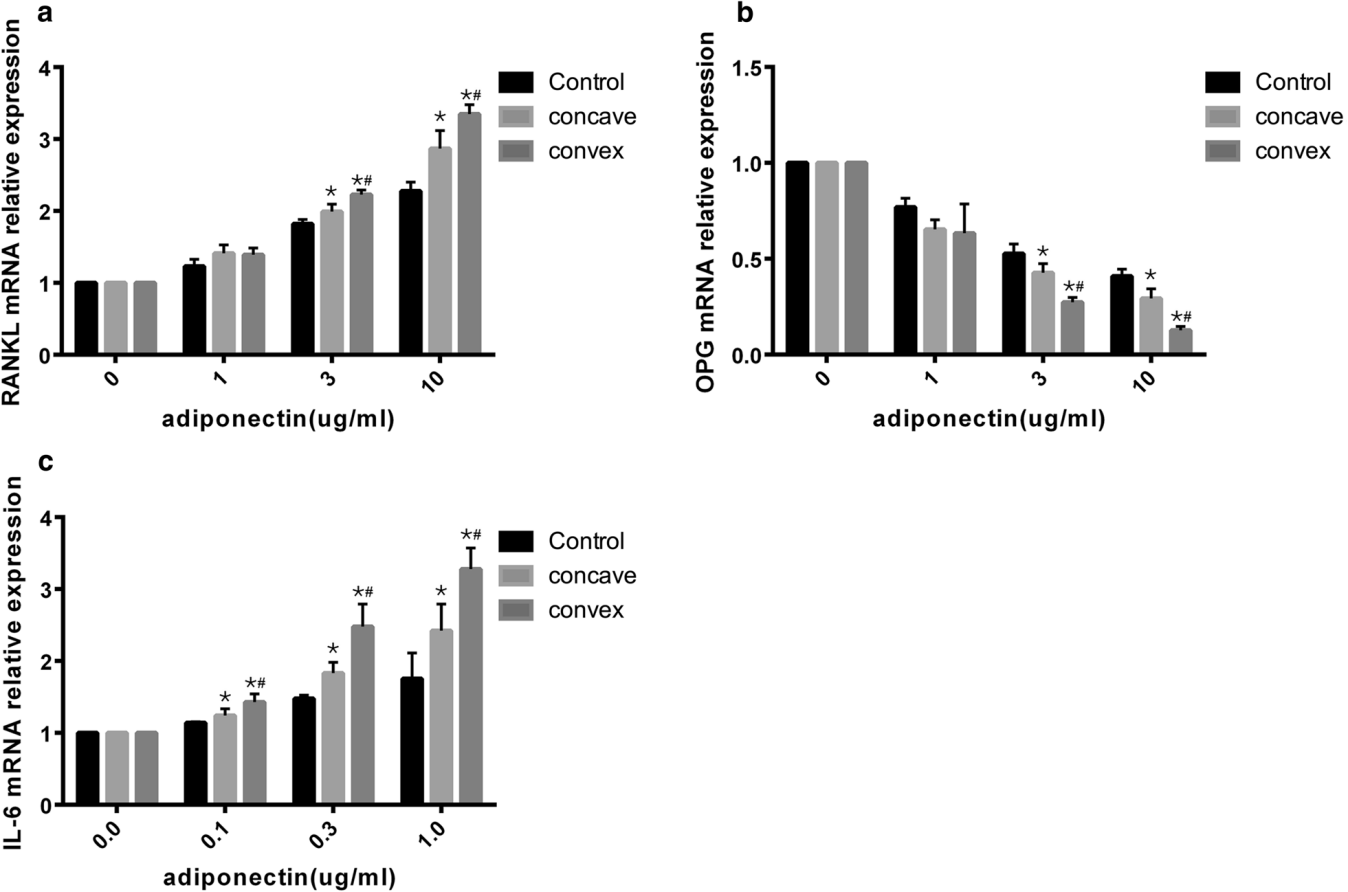
Effect of adiponectin on primary cells. Effect of adiponectin on human primary osteoblast. **a**, **b** Primary osteoblast were exposed to 1–10 μg/mL adiponectin for 48 h. RANKL and OPG mRNA expression were determined by quantitative real-time PCR. The Y axis represents the fold change and the expression of RANKL and OPG at 0 μg/mL were set to 1.0. Effect of adiponectin on human primary chondrocyte. **c** Primary chondrocyte were exposed to 0.1–1 μg/mL adiponectin for 24 h. IL6 mRNA expression were determined by quantitative real-time PCR. The Y axis represents the fold change and the expression of IL6 at 0 μg/mL were set to 1.0. One way ANOVA was use to tested, when p value > 0.05, there was no significant difference in all the groups; when p value < 0.05, then using bonferroni correction to test each two groups. *p < 0.0167 Bonferroni correction test between controls and AIS concave. Bonferroni correction test between controls and AIS convex. ^#^p < 0.0167 Bonferroni correction test between AIS concave and AIS convex

Next, we assessed the direct effect of adiponectin on primary chondrocytes and osteoblasts. Primary cells were isolated from the facet joints of 5 patients and 5 age-matched patients with lumbar intervertebral disc herniation as controls (Additional file 5: Table S5). The identification of cell staining was in Additional file 6: Figure S1 and Additional file 7: Figure S2. For AIS and control primary osteoblasts, treatment with adiponectin (0–10 µg/mL) for 48 h induced RANKL mRNA in a concentration-dependent manner (Fig. 4a). Moreover, the amount of RANKL expression in AIS was higher than that in the control group. Another bone protective factor (opg) was inhibited in a dose-dependent manner in the primary osteoblasts of both groups; opg in the AIS group was decreased more than that in the control group. Moreover, the convex side showed more acute effects than the concave side. For AIS and control primary chondrocytes, treatment with adiponectin (0–1 µg/mL) for 24 h induced IL-6 mRNA in a concentration-dependent manner (Fig. 4b). However, the amount of IL-6 expression in the AIS group was higher than that in the control group, and the IL-6 levels on the convex side were significantly higher than those on the concave side.

## Discussion

Numerous studies have reported causes of AIS, including (but not limited to) genetic, hormonal, neuromuscular, and neurodevelopmental abnormalities; environmental or motor-control factors; lifestyle; and bone metabolism. However, Schlosser et al. [28] recently performed a review comprising different proposed theories of AIS and found that only etiologies related to impaired gait control and decreased bone mineral density showed moderate evidence with respect to AIS. Other etiological factors, such as asymmetric somatosensory evoked potentials and reduced trunk muscle strength, showed weak evidence for AIS, indicating that decreased bone mineral density plays an important role in AIS. Bone mass increases rapidly in childhood, especially in adolescence. During this period, many factors can affect this process, especially hormones. In AIS patients, previous studies have indicated that leptin, estrogen, melatonin and ghrelin are involved in the low bone mass [14, 15]. Recently, another hormone, adiponectin, an adipose-specific collagen-like protein that is highly expressed in adipocytes, was proven to play a role in AIS. In a population-based prospective study on UK corhort, Clark et al. [29] measured plasma adiponectin of 7298 children at 10 and 15 years and they found that a weakly positive association between adiponectin level at age 10 years and scoliosis. In another study, Jiang et al. [30] performed RNA-sequencing on paravertebral muscle from AIS patients and found that asymmetric expression of adipoQ (adiponectin) in concave/convex side. However, the function of adiponectin involved in the AIS was still unknown. Because previous study reported adiponectin was often correlated with low bone mass and low body mass which is the common phenomena in AIS, we recruited normal bone mass AIS, osteopenia AIS and control to measure the plasma adiponectin level. And the resulted showed that the level of adiponectin in osteopenia AIS was significant higher than normal bone mass AIS and control. Although patients with normal bone mass AIS showed 1.34 times higher plasma levels than the control subjects did, there was no significant difference (p = 0.063). Combined with previous studies, the present results led us to assume that adiponectin played a more important role in AIS osteopenia than in normal-bone-mass AIS. In addition, we also observed that osteopenia AIS has lower BMI and larger Cobb angle, which is consisted with previous studies.

So in the next part, we tried to find the reason of higher adiponectin in AIS osteopenia. The plasma adiponectin level is influenced by many different factors. For example, Kazumi et al. [31] found that among young men, individuals with a high-normal blood pressure person had lower serum adiponectin levels. Hanley [32] reported that adiponectin concentrations were significantly lower among men versus women and among diabetic patients versus individuals with normal glucose tolerance. Furthermore, the adiponectin gene sequence reveals a large number of allele SNPs that may affect the plasma level. Rs12637534, rs1648707/rs6810075 and rs17366568 reduce adiponectin levels, whereas rs10937273, rs16861209/rs822387, rs822395, rs3774261, rs6444175 and rs17373414 are associated with higher adiponectin levels [33]. In our study, because the age and gender distribution showed no significant difference, we selected nine SNPs that were proven to affect the plasma adiponectin concentration for a genotypic analysis. We recruited 200 osteopenia AIS, 209 normal bone mass AIS and 206 controls for genotypic analyses. The results showed in rs7639352, T allele (41.75%) were significantly more frequent in the osteopenia AIS group than normal bone mass AIS (31.3%) and control group (25.7%). Other SNPs showed no significant difference. In a previous study, Dhillon et al. and Heid et al. reported that the rs7639352 T allele was associated with high plasma adiponectin levels in different populations, which was consistent with our results, indicating that a high adiponectin concentration in osteopenia AIS may be due to the gene variation [34, 35].

However, the exact way of adiponectin participating in AIS osteopenia were unknown. We took surgical specimen as the object of the study. Unbalanced spinal growth between left and right side, anterior and posterior column is proven to be important roles on development and progression of AIS [36]. Dickson et al. [37] reported that in IS, coronal and sagittal plane present asymmetry growth, whereas Stilwell speculated that the asymmetrical bone growth was the main etiology of scoliosis [38]. Another study showed that in AIS patients, convex and concave side demonstrated difference in histological grades and cellular activity [39]. However, in clinical, it is difficult to obtain the anterior column tissues and no one had evaluated the difference in bone mass in bilateral facet joints in osteopenia AIS patients. So in the present study, we selected concave and convex side facet joint to study the asymmetric situation. Micro-CT revealed that the BV/TV and Tb.N of the convex side were significantly lower than those of the concave side. And Tb.Sp was significantly higher in convex side than in concave side. BV/TV is an index that reflects the amount of bone mass, whereas Tb.N, Tb.Sp, and Tb.Th better explain changes in the bone microarchitecture [40]. Bone growth has been reported to increase Tb.Th, BV/TV, and Tb.N but decrease Tb.Sp. Our results suggest that the convex side has a lower bone volume than the concave side which is consistent with Wolff’s law, that is, bone formation will increase in areas of compression and decrease in areas of tension. Although previous studies have reported that the convex side shows a higher bone volume than the concave side in a rodent model of scoliosis [41], we assumed that this discrepancy might be because animal models cannot fully reflect the pathophysiology of AIS. In addition, the qPCR, western blotting and immunochemistry also confirmed the imbalance between concave and convex side. Higher RANKL/OPG, higher RANK and more osteoclast number were observed in convex side, which may lead to bilateral asymmetric bone mass in concave and convex side. To obtain a better understanding of the exact mechanisms in AIS, primary osteoblasts were obtained from facet joints. Osteoblasts were exposed to a graded concentration of adiponectin (from 0 to 10 µg/mL) for 48 h. Adiponectin induced RANKL release and suppressed OPG release in a dose-dependent and time-dependent manner. This finding was consistent with that of the previous study. In vitro, in primary adult osteoblasts, adiponectin-induced RANKL and OPG change in a dose-dependent manner from 0 to 30 µg/mL in a time-dependent manner through the MAPK-p38 signaling pathway [20]. Although the graded concentrations are not exactly the same, we assume that these results might be related to the donor sites and ages. In vivo, 12-week-old transgenic Adipo^−/−^ mice exhibit higher OPG levels and lower RANKL levels than wild-type (WT) mice [42].

Additionally, it is well documented that the production of local and circulating pro-inflammatory cytokines, including TNF-α, IL6, and IL10, plays an important role in the occurrence and development of osteopenia by inhibiting osteoblast function and promoting osteoclast differentiation [43]. In osteoblasts, these cytokines suppress osteoblast differentiation through downregulating the BMP and TGF-beta signaling pathway [44]. In osteoclasts, pro-inflammatory cytokines may stimulate RANKL release, which promotes osteoclast function [45]. Moreover, adiponectin has been proven to be involved in promoting pro-inflammatory cytokines in chronic inflammatory diseases such as rheumatoid arthritis and osteoarthritis. For instance, Koskinen et al. [46] found that in osteoarthritis, plasma adiponectin levels are associated with the bone destruction grade, and adiponectin enhances the release of NO, IL-6, MMP-1 and MMP-3 from primary chondrocytes through the MAPK signaling pathway. In another study, adiponectin was found to cause concentration- and time-dependent IL6 production in rheumatoid-arthritis synovial fibroblasts and osteoarthritis synovial fibroblasts via an adipoR1 receptor, AMPK, p38 and NF-kB pathway [47]. In present study, we detected the expression of inflammatory factors in cartilage. The results showed that IL-6 is highly expressed in cartilage in AIS osteopenia. Furthermore, we observed the asymmetric expression of IL-6 in bilateral facet joints. The IL6 expression is significant higher in convex side than concave side. We also extracted primary chondrocyte and exposed under adiponectin gradient concentration (0.1–1 μg/mL). Cell experiments demonstrated that adiponectin induced IL-6 production in and concentration-dependent manner. This result indicated that adiponectin was involved in the asymmetric bone mass in bilateral through the release of IL6 from AIS chondrocytes.

Two high-affinity cell surface receptors for adiponectin, adipoR1 and adipoR2, have been identified in human tissues. Previous research found that adipoR1 is abundantly expressed in skeletal muscle and has a high affinity for globular adiponectin, whereas adipoR2 is highly expressed in the liver and has an intermediate affinity for globular and full-length adiponectin. Although the two receptors were found in bone-forming cells, previous studies have revealed that adipoR1 plays a main role in signal transduction [20, 21, 47]. In AIS facet joints, we found that ADR1 significant higher than normal in osteoblasts and chondrocytes, respectively. And ADR2 showed no significant difference. Furthermore, asymmetric expression of ADR1 was observed in convex and concave side. The results indicated that the high expression of adiponectin receptor 1 may cause it to receive more signals and amplify its biological function. Therefore, compared with normal primary cells, AIS primary cells exhibit more acute reactions and convex-side primary cells released more RANKL and IL6 cytokines at the same concentration.

**Fig. 5:**
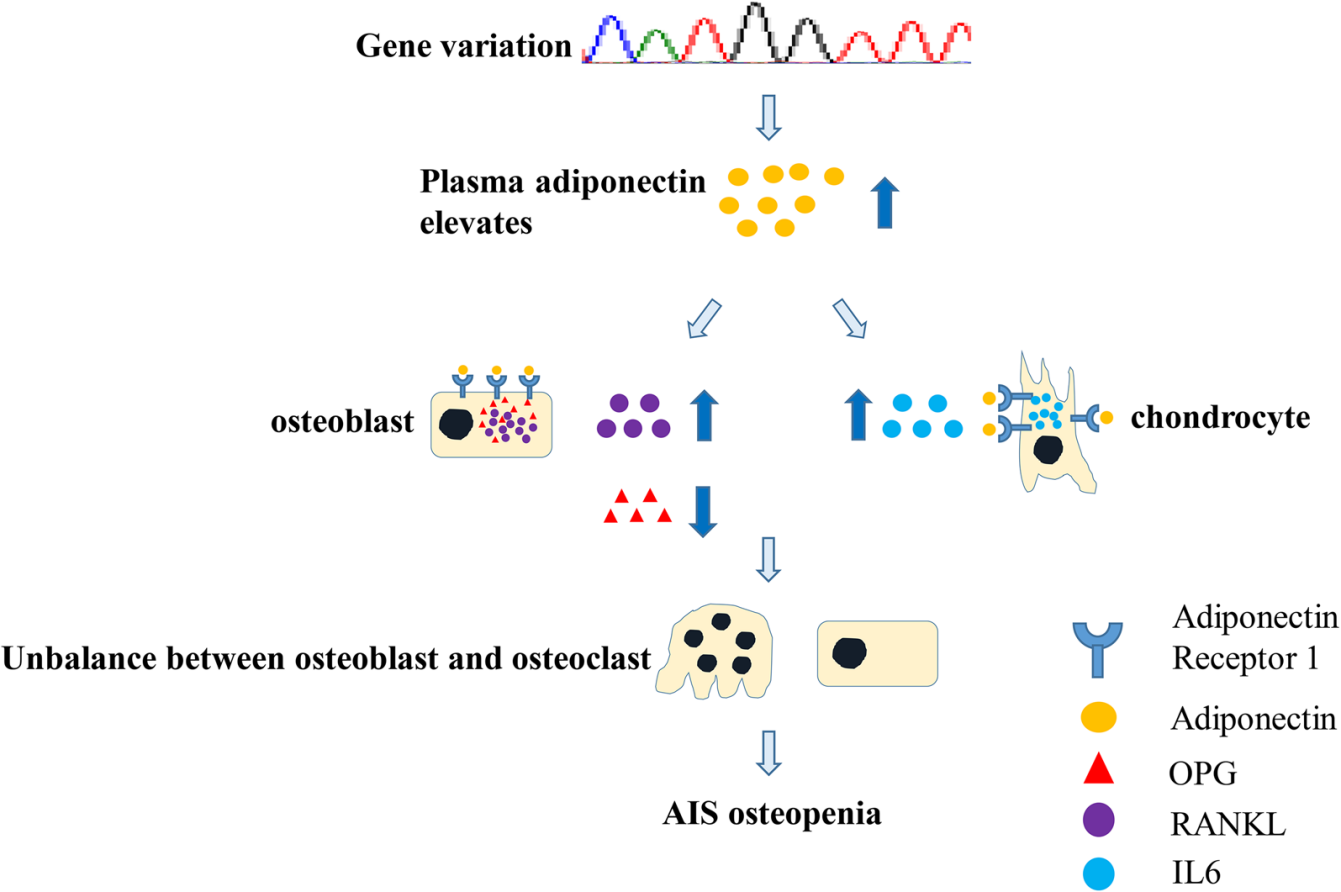
The function of adiponectin in AIS osteopenia. Gene variation leads to the increase in plasma adiponectin which exerts its negative effect on bone metabolism via RANKL/OPG and IL6 pathway in primary osteoblast and chondrocyte in AIS osteopenia

In our study, plasma adiponectin significantly elevated in AIS osteopenia and this increase maybe due to the gene variation. Additionally, adiponectin significantly stimulated increases in RANKL and IL6 in AIS primary cell and the increases induced by adiponectin appeared to be higher than in normal primary cell. These factor can promote osteoclasts activation, differentiation and prevent osteoclast from apoptosis which contributed to the AIS osteopenia (Fig. 5). These results, to our knowledge, is the first report of adiponectin function in AIS osteopenia. However, there are some limitations that should be considered. One is the limited sample size of the recruited patients. Patients with lower Cobb angles were excluded because they do not need surgical therapy. The second limitation is that the primary cells were extracted from facet joints, which may not represent the general trend. The third limitation is that all experiments were performed in vitro, and in vivo experiments may demonstrate different results.

## Conclusion

In conclusion, our findings provide new insights into AIS osteopenia. We found that in AIS osteopenia, high plasma adiponectin levels which may due to the gene variation may affect bone mass through the modulation of RANKL/OPG in osteoblasts and IL6 release in chondrocytes by binding to adipoR1. These results suggest that adiponectin plays an important role in AIS osteopenia.

## Additional files

**Additional file 1: Table S1.** Clinical data of SNP subjects.

**Additional file 2: Table S2.** Clinical data of Micro-CT subjects.

**Additional file 3: Table S3.** Clinical data of Real-time quantitative PCR and Western blotting subjects.

**Additional file 4: Table S4.** Clinical data of Immunochemistry subjects.

**Additional file 5: Table S5.** Clinical data of cell experiment subjects.

**Additional file 6: Figure S1.** Alizarin red staining. P2 generation osteoblast was stained.

**Additional file 7: Figure S2.** Toluidine blue staining. P2 generation chondrocyte was stained.

## Abbreviations

AIS: adolescent idiopathic scoliosis
BMI: body mass index
BMD: bone marrow density
AdipoR1: adiponectin receptor 1
AdipoR2: adiponectin receptor 2
adipoQ: adiponectin
SNP: single nucleotide polymorphism
ELISA: enzyme-linked immunosorbent assay
iMLDR: improved multiplex ligation detection reaction
PVDF: polyvinylidene difluoride
AOD: average optical density
