# Supplementary material for: Adiponectin regulates bone mass in AIS osteopenia via RANKL/OPG and IL6 pathway
Source: J Transl Med. 2019 Feb 28;17:64. doi: 10.1186/s12967-019-1805-7 (PMC6396498; doi:10.1186/s12967-019-1805-7)
Supplement: Supplementary file 1 — Additional file 1: Table S1. Clinical data of SNP subjects. [file 12967_2019_1805_MOESM1_ESM.doc]

**Table S1** Clinical data of SNP subjects

| Items | AIS group | | Control | P value |
| --- | --- | --- | --- | --- |
|  | Osteopenia | Normal bone mass |  |  |
| Number(male/female) | 200(83/117) | 209(99/110) | 206 (105/101) | ＞0.05 |
| Age(years) | 15.35±2.53 | 15.05±1.95 | 15.52±2.1 | ＞0.05 |
| LS Z SCORE | -1.45±0.59 | -0.18±0.36 | -0.15±0.65 | **＜0.05** |
| Main curve cobb angle (°) | 27.9±10.5 | 23.65±7.6 |  | **＜0.05** |
| Lenke classification |  |  |  |  |
| I | 75 | 72 |  |  |
| II | 13 | 13 |  |  |
| III | 10 | 17 |  |  |
| IV | 11 | 9 |  |  |
| V | 61 | 75 |  |  |
| VI | 30 | 23 |  |  |
