# Supplementary material for: Adiponectin regulates bone mass in AIS osteopenia via RANKL/OPG and IL6 pathway
Source: J Transl Med. 2019 Feb 28;17:64. doi: 10.1186/s12967-019-1805-7 (PMC6396498; doi:10.1186/s12967-019-1805-7)
Supplement: Supplementary file 2 — Additional file 2: Table S2. Clinical data of Micro-CT subjects. [file 12967_2019_1805_MOESM2_ESM.docx]

**Table S2 Clinical data of Micro-CT subjects**

| Items | Sex | Age(yrs) | Height(m) | Weight(kg) | BMI(kg/m^2^) | BMC(g) | BMD(g/m^2^) | LS Z Score | Lenke Classification | apical vertebra |
| --- | --- | --- | --- | --- | --- | --- | --- | --- | --- | --- |
| Patient 1 | Male | 14 | 1.53 | 31 | 13.24 | 41.80 | 0.762 | -0.8 | Lenke 3 | T9/L2 |
| Patient 2 | Female | 13 | 1.6 | 46 | 17.96 | 35.47 | 0.773 | -1.0 | Lenke 5 | L2 |
| Patient 3 | Female | 15 | 1.6 | 60 | 23.44 | 35.23 | 0.908 | -0.6 | Lenke 1 | T8 |
| Patient 4 | Female | 14 | 1.55 | 40 | 16.65 | 33.76 | 0.675 | -2.3 | Lenke 1 | T8 |
| Patient 5 | Female | 13 | 1.58 | 37 | 14.82 | 41.98 | 0.725 | -1.3 | Lenke 1 | T9 |
