# Supplementary material for: Adiponectin regulates bone mass in AIS osteopenia via RANKL/OPG and IL6 pathway
Source: J Transl Med. 2019 Feb 28;17:64. doi: 10.1186/s12967-019-1805-7 (PMC6396498; doi:10.1186/s12967-019-1805-7)
Supplement: Supplementary file 3 — Additional file 3: Table S3. Clinical data of Real-time quantitative PCR and Western blotting subjects. [file 12967_2019_1805_MOESM3_ESM.doc]

**Table S3 Clinical data of Real-time quantitative PCR and Western blotting subjects**

| Items | AIS group | Control | P value |
| --- | --- | --- | --- |
| Number(male/female) | 30(11/19) | 30(15/15) | ＞0.05 |
| Age(years) | 15.30±3.05 | 15.00±2.56 | ＞0.05 |
| LS Z SCORE | -1.95±0.36 | -0.11±0.37 |  |
| FN Z SCORE | -2.25±0.68 | -1.00±0.78 |  |
| Main curve cobb angle (°) | 50.72±10.39 |  |  |
| Lenke classification |  |  |  |
| I | 10 |  |  |
| II | 1 |  |  |
| III | 2 |  |  |
| IV | 3 |  |  |
| V | 9 |  |  |
| VI | 5 |  |  |
