# Supplementary material for: Adiponectin regulates bone mass in AIS osteopenia via RANKL/OPG and IL6 pathway
Source: J Transl Med. 2019 Feb 28;17:64. doi: 10.1186/s12967-019-1805-7 (PMC6396498; doi:10.1186/s12967-019-1805-7)
Supplement: Supplementary file 4 — Additional file 4: Table S4. Clinical data of Immunochemistry subjects. [file 12967_2019_1805_MOESM4_ESM.doc]

**Table S4 Clinical data of** **Immunochemistry subjects**

| Items | AIS group | Control | P value |
| --- | --- | --- | --- |
| Number(male/female) | 22(8/14) | 15 (10/5) | ＞0.05 |
| Age(years) | 13.63±2.90 | 15.13±2.77 | ＞0.05 |
| LS Z SCORE | -2.54±0.30 | 0.03±0.35 |  |
| FN Z SCORE | -2.79±0.92 | -0.79±0.83 |  |
| Main curve cobb angle (°) | 50.72±10.86 |  |  |
| Lenke classification |  |  |  |
| I | 6 |  |  |
| II | 0 |  |  |
| III | 4 |  |  |
| IV | 2 |  |  |
| V | 9 |  |  |
| VI | 1 |  |  |
