# Supplementary material for: Adiponectin regulates bone mass in AIS osteopenia via RANKL/OPG and IL6 pathway
Source: J Transl Med. 2019 Feb 28;17:64. doi: 10.1186/s12967-019-1805-7 (PMC6396498; doi:10.1186/s12967-019-1805-7)
Supplement: Supplementary file 5 — Additional file 5: Table S5. Clinical data of cell experiment subjects. [file 12967_2019_1805_MOESM5_ESM.docx]

**Table S5 Clinical data of cell experiment subjects**

| Items | Sex | Age  (yrs) | Height  (m) | Weight  (kg) | BMI  (kg/m^2^) | BMC  (g) | BMD  (g/m^2^) | LS Z Score | Lenke Classification | apical vertebra |
| --- | --- | --- | --- | --- | --- | --- | --- | --- | --- | --- |
| Patient 1 | Male | 14 | 1.65 | 42 | 15.4 | 38.17 | 0.7 | -1.2 | Lenke 1 | T8 |
| Patient 2 | Male | 15 | 1.61 | 41 | 15.8 | 41.04 | 0.725 | -1.3 | Lenke 1 | T7 |
| Patient 3 | Female | 13 | 1.67 | 47 | 16.9 | 41.08 | 0.785 | -0.1.1 | Lenke 5 | L3 |
| Patient 4 | Female | 16 | 1.62 | 46 | 17.5 | 49.27 | 0.88 | -1 | Lenke 5 | L2 |
| Patient 5 | Female | 16 | 1.58 | 45 | 18.02 | 41.64 | 0.802 | -0.7 | Lenke 1 | T6 |
| Control 1 | Male | 15 | 1.65 | 50 | 18.37 | 49.31 | 0.891 | -0.2 |  |  |
| Control 2 | Male | 16 | 1.66 | 56 | 20.32 | 53.69 | 0.93 | -0.4 |  |  |
| Control 3 | Male | 17 | 1.78 | 60 | 18.94 | 63.74 | 1.04 | 0.1 |  |  |
| Control 4 | Female | 14 | 1.6 | 50 | 19.53 | 50.37 | 0.91 | -0.3 |  |  |
| Control 5 | Female | 14 | 1.7 | 53 | 18.34 | 56.17 | 0.92 | 0.1 |  |  |
