# Supplementary figures and images for: Adiponectin regulates bone mass in AIS osteopenia via RANKL/OPG and IL6 pathway
Source: J Transl Med. 2019 Feb 28;17:64. doi: 10.1186/s12967-019-1805-7 (PMC6396498; doi:10.1186/s12967-019-1805-7)

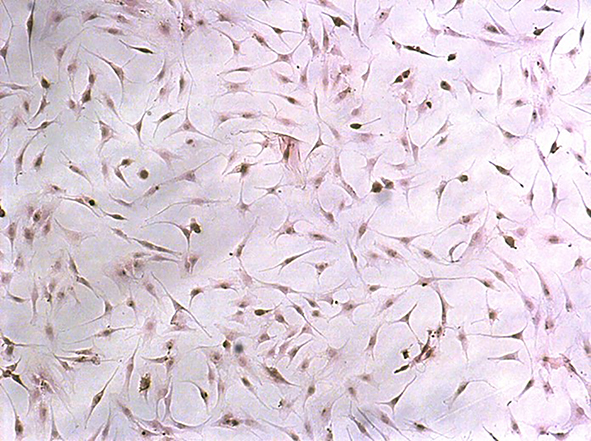

Supplement: Supplementary file 6 — Additional file 6: Figure S1. Alizarin red staining. P2 generation osteoblast was stained. [file 12967_2019_1805_MOESM6_ESM.tif]

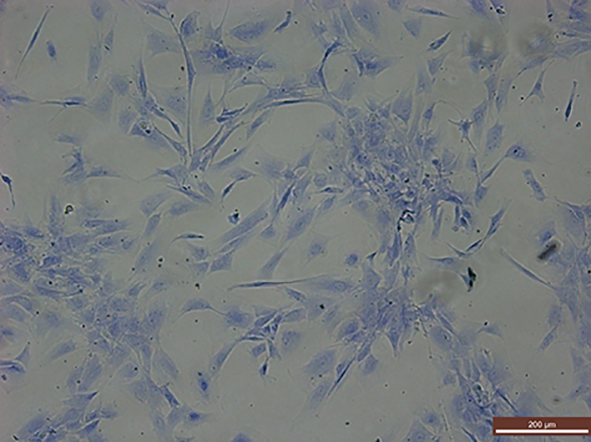

Supplement: Supplementary file 7 — Additional file 7: Figure S2. Toluidine blue staining. P2 generation chondrocyte was stained. [file 12967_2019_1805_MOESM7_ESM.tif]
